# Epidemiological Trends of Candidemia and the Impact of Adherence to the Candidemia Guideline: Six-Year Single-Center Experience

**DOI:** 10.3390/jof7040275

**Published:** 2021-04-06

**Authors:** Jong Hun Kim, Jin Woong Suh, Min Ja Kim

**Affiliations:** 1 Department of Internal Medicine, Division of Infectious Diseases, Korea University College of Medicine, Seoul 02841, Korea; sunthes@naver.com (J.W.S.); macropha@korea.ac.kr (M.J.K.); 2 CHA Bundang Medical Center, Department of Internal Medicine, Division of Infectious Diseases, CHA University, Seongnam 13496, Korea

**Keywords:** candidemia, epidemiology, EQUAL Candida score, mortality

## Abstract

This study aimed to investigate the epidemiology of candidemia and evaluate the impact of adherence to the candidemia guideline defined by the European Confederation of Medical Mycology Quality of Clinical Candidemia Management (EQUAL) Candida score. Adult candidemia patients ≥ 19 years diagnosed at a tertiary care hospital in the Republic of Korea from 2013 to 2018 were enrolled (period 1 2013–2015, period 2 2016–2018). There was a total of 223 patients. The annual incidence of candidemia increased from 0.43 to 1.33 cases per 1000 admissions between 2013 and 2018, *p* < 0.001. A significant increase of fluconazole-resistant *C. parapsilosis* candidemia was noted in period 2 (35.3%) when compared to period 1 (0.0%), *p* = 0.020. The 30-day mortality rate was not different between period 1 and 2 (43.5% vs. 48.1%, *p* = 0.527). Multivariate analysis revealed that a Charlson comorbidity index score ≥ 4, neutropenia, duration of hospital stay ≥ 21 days before candidemia diagnosis, septic shock, mycological failure, and EQUAL Candida score < 15 were significantly associated with 30-day mortality. An increase in the incidence of candidemia and fluconazole resistance in the non-*albicans Candida* species over time was observed. Disease severity, comorbidities, and lower adherence to the candidemia guideline were associated with mortality.

## 1. Introduction

*Candia* species may cause invasive disease, and the most common form of invasive candidiasis is candidemia [1]. The incidence of candidemia in the hospital setting has increased over recent decades [2] and is associated with significant morbidity and mortality [3]. Although *C. albicans* continues to be the most frequent pathogen, candidemia caused by non-*albicans Candida* is increasing globally, including the Asia-Pacific region [4,5]. In the Republic of Korea (ROK), an increase of non-*albicans Candida* candidemia has been reported recently [6,7]. Also, there has been an increase in the aging population with comorbidities in the ROK [8], suggesting there may be higher incidence rates of candidemia among adult patients in the hospital setting in the ROK. Moreover, resistance to antifungal agents is an emerging problem associated with an increasing use of antifungal agents [9]. In the ROK, fluconazole or amphotericin B was mainly used for the treatment of candidemia until the approval of echinocandins as primary treatment for severe candidiasis by the National Health Insurance Service (NHIS) in 2014. Following the approval of echinocandin use by the NHIS, the use of echinocandins for candidemia treatment has increased in the ROK [10]. Furthermore, the current candidemia guideline by the Infectious Diseases Society of America (IDSA) published in 2016 [1] recommends an echinocandin as the primary initial antifungal agent for candidemia treatment. Proper management of candidemia is critical, and adherence to the guideline may improve outcomes as a recent study showed that greater guideline adherence was associated with survival of candidemia patients [11]. However, there have been few data regarding epidemiological trends of candidemia and the impact of adherence to the candidemia guideline in the ROK after approval of echinocandin use by the NHIS and publication of the guideline. Therefore, this study aimed to investigate the epidemiology, clinical characteristics, adherence to the guideline, and outcomes of candidemia among hospitalized adult patients in the ROK in recent years 2013–2018.

## 2. Materials and Methods

### 2.1. Study Design and Population

A retrospective study of adult patients admitted at a tertiary care hospital (Korea University Anam Hospital, Seoul, Korea) from 2013 to 2018 was conducted. Inclusion criteria were: (1) adult patients ≥ 19 years old; (2) patients diagnosed with candidemia. Exclusion criteria were: (1) patients without candidemia; (2) patients < 19 years old. Patients’ demographics and clinical data, including underlying comorbidities, clinical conditions at the time of candidemia diagnosis, candidemia management, and outcomes, were collected for period 1 (2013–2015) and period 2 (2016–2018). This study was approved by the institutional review board at the Korea University Anam Hospital (IRB Number 2018AN0440). Informed consent was not required due to the retrospective design of the study.

### 2.2. Definition

A case of candidemia was defined as at least one positive peripheral blood culture for the growth of *Candida* species obtained from an adult hospitalized patient ≥ 19 years old. Antifungal treatment was initiated if *Candida* species were identified from the blood culture at the discretion of treating physicians. Follow-up blood cultures after initiation of antifungal treatment were performed every day or every other day until candidemia was cleared from the blood culture. Identification and antifungal susceptibility of *Candida* spp. from blood culture were performed using the BacT/ALERT^®^ 3D Microbial Detection System (bioMérieux, Inc., Durham, NC, USA) and the automated Vitek^®^ 2 Yeast Biochemical Card (bioMérieux, Inc., Durham, NC, USA). Antifungal susceptibility testing results were interpreted according to Clinical and Laboratory Standards Institute (CLSI) breakpoints as recommended in the guideline [1]. The Charlson comorbidity index was calculated to assess the impact of comorbidities. For each patient, clinical conditions were collected as follows: mechanical ventilation, urinary catheter, central venous catheter, parenteral nutrition, hemodialysis, steroid use, neutropenia, chemotherapy, duration of hospital stay before candidemia diagnosis, recent surgery in the current admission, previous admission to intensive care units (ICUs) within three months, previous use of antibiotic within a month, source of candidemia, antifungal treatment, length of hospital stay after diagnosis of candidemia. Clinical conditions were defined as follows: (1) neutropenia as an absolute neutrophil count of <500 cells/mm^3^; (2) steroid use as systemic steroid (≥20 mg/day of prednisone equivalent) use; (3) chemotherapy as use of antimetabolites; (4) septic shock as adapted from the third International Consensus Definitions for Sepsis and Septic Shock (Sepsis-3) [12].

Adherence to the candidemia guideline [1] was measured by calculation of The European Confederation of Medical Mycology Quality of Clinical Candidemia Management score (EQUAL Candida score) [13]. The source of candidemia was classified based on clinical evidence of infection using the definition from a previous study [14]. Outcomes of candidemia were assessed in the followings: (1) mycological response defined as eradication of candidemia resulted in negative blood culture (mycological failure defined as a failure to eradicate candidemia); (2) 30-day mortality defined as death within 30 days after the first positive blood culture for candidemia.

### 2.3. Statistical Analysis

The incidence of candidemia was measured as the number of candidemia cases per 1000 hospital admissions. A Poisson regression was used for trend analysis of the annual incidence of candidemia. Categorical variables were analyzed by the Pearson’s Chi-square test of Fisher’s exact test. The Mann–Whitney test was used for continuous variables. Variables with a *p*-value < 0.1 on comparison analysis were included in a multiple logistic regression analysis to determine risk factors associated with 30-day mortality. The Kaplan–Meier curve was used for survival analysis between candidemia patients with EQUAL Candida score ≥ 15 and EQUAL Candida score < 15. A *p* value < 0.05 was considered to be statistically significant. SPSS v.23.0 for Windows (SPSS Inc., Chicago, IL, USA) was used for statistical analyses.

## 3. Results

### 3.1. Patient Population and Clinical Characteristics Stratified by Study Periods

**Figure 1:**
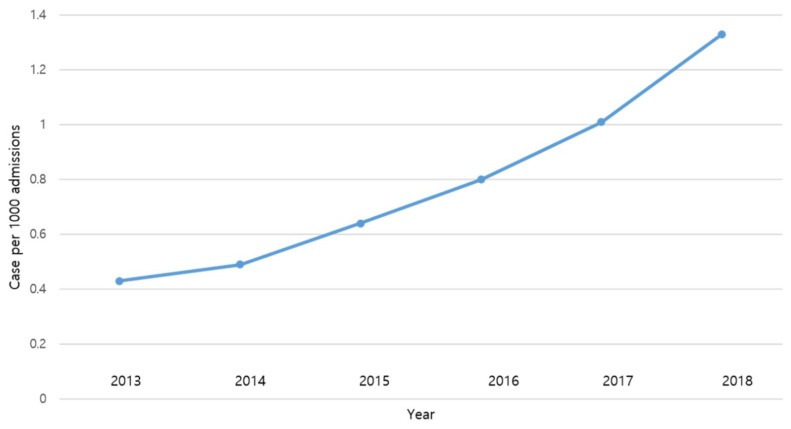
Incidence of candidemia 2013–2018 (Figure modified from Kim et al. J Mycol Med. 2020 Dec 1;31(1):101102, with permission of Elsevier. This article was published in Journal de Mycologie Médicale, Volume 31, Issue 1, Kim et al., Prevalence and risk factors for endogenous fungal endophthalmitis in adult patients with candidemia at a tertiary care hospital in the Republic of Korea over 13 years, Copyright Elsevier (2021)).

During the study period, there were 223 adult patients diagnosed with candidemia who were enrolled in the study after the application of the inclusion and exclusion criteria. The median age of these 223 patients was 71 years with an interquartile range (IQR) of 60–79 years. There were 127 males (57.0%). The incidence of candidemia significantly increased throughout the study period (0.43 cases per 1000 admissions for 2013, 0.49 cases per 1000 admissions for 2014, 0.64 cases per 1000 admissions for 2015, 0.80 cases per 1000 admissions for 2016, 1.01 cases per 1000 admissions for 2017, and 1.33 cases per 1000 admissions) with the annual incidence rate ratio of 1.267 (95% confidence interval (CI) 1.167–1.376, *p* < 0.001). These are shown in Figure 1.

The patients were categorized into two groups (candidemia diagnosed in period 1 (2013–2015) and period 2 (2016–2018)). There were more older patients with age ≥ 75 years in period 2 than in period 1 (43.5% vs. 26.1%, *p* = 0.013). Regarding underlying comorbidities, there was no significant difference between the two groups. These underlying comorbidities were Charlson comorbidity index, heart disease, lung disease, kidney disease, liver disease, diabetes mellitus, neurological disease, and malignancy. However, there were more patients with mechanical ventilation (34.4% vs. 18.8%, *p* = 0.019), urinary tract catheterization (81.2% vs. 59.4%, *p* = 0.001), parenteral nutrition (95.5% vs. 87.0%, *p* = 0.045), and longer duration of hospital stay before candidemia diagnosis (median 21 days vs. 13 days, *p* = 0.046) in the period 2 than in the period 1. Also, there was a non-significant trend of more patients with the previous admission to the ICUs within three months in period 2. Clinical conditions of steroid use (58.0% vs. 37.7%, *p* = 0.005), neutropenia (14.5% vs. 5.8%, *p* = 0.032), and recent surgery in the current admission (46.4% vs. 18.8%, *p* < 0.001) were more frequently observed in the period 1 than in the period 2. Regarding the source of candidemia, the most common source was the central venous catheter both in period 1 and period 2 without a difference. However, urinary tract related candidemia was more common (7.8% vs. 0.0%, *p* = 0.020) in period 2 while others or unknown source related candidemia was more common in period 1 (40.6% vs. 15.6%, *p* < 0.001). Although the duration of antifungal treatment was similar, the use of antifungal treatment was significantly different between period 1 and period 2. Fluconazole was more frequently used in period 1 (55.8% vs. 23.8%, *p* < 0.001) while there was more use of echinocandins in period 2 (75.4% vs. 34.6%, *p* < 0.001). There were higher EQUAL Candida scores in period 2 for overall patients as well as patients with and without the central venous catheter. Regarding 30-day mortality after diagnosis of candidemia, there was no significant difference between period 1 and period 2 (43.5% vs. 48.1%, *p* = 0.527) (Table 1).

### 3.2. Analysis of Risk Factors for Mortality

**Figure 2:**
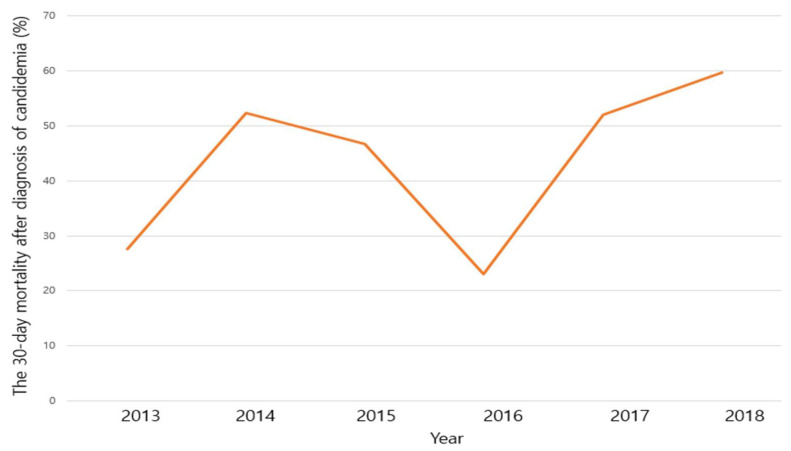
The 30-day mortality after diagnosis of candidemia 2013–2018.

Overall, the 30-day mortality after diagnosis of candidemia during the study period was 46.6%. Although annual rate of the 30-day mortality was different for each study year (27.5% for 2013, 52.4% for 2014, 46.7% for 2015, 23.1% for 2016, 52.1% for 2017, 59.7% for 2018), the annual 30-day mortality rate ratio did not show statistical significance (1.116, 95% CI 0.983–1.266, *p* = 0.090). These are shown in Figure 2.

Based on the 30-day mortality after diagnosis of candidemia, patients were categorized into the two groups (survivor and non-survivor). The distribution of age and sex was similar between the two groups. Regarding underlying comorbidities, there was a trend of more patients with a Charlson comorbidity index ≥ 4 in the non-survivor group than in the survivor group (47.1% vs. 35.3%, *p* = 0.073). Furthermore, non-survivors had significantly more mechanical ventilation (40.4% vs. 20.2%, *p* = 0.001), central venous catheter (78.8% vs. 65.5%, *p* = 0.028), hemodialysis (29.8% vs. 12.6%, *p* = 0.002), steroid use (52.9% vs. 36.1%, *p* = 0.012), and neutropenia (15.4% vs. 2.5%, *p* = 0.001) when compared with survivors. Also, non-survivors had a longer duration of hospital stay before candidemia diagnosis (29 days vs. 12 days, *p* < 0.001) and more previous admission to ICUs within three months (46.2% vs. 30.3%, *p* = 0.014). Regarding the source of candidemia, central venous catheter-related candidemia was more common in the non-survivor group (65.4% vs. 52.1%, *p* = 0.045). The distribution of *Candida* species was significantly different. *C. tropicalis* (30.8% vs. 16.8%, *p* = 0.014) and *C. guillermondii* (3.8% vs. 0.0%, *p* = 0.046) were more prevalent in the non-survivor group. In comparison, there were more patients with *C. parapsilosis* (26.9% vs. 13.5%, *p* = 0.013) in the survivor group. There was a nonsignificant trend of higher mortality rate in patients with fluconazole resistant *C. parapsilosis* than in patients with fluconazole susceptible *C. parapsilosis* (41.7% vs. 26.5%, *p* = 0.325). The proportion of the patients who received antifungal treatment was significantly higher in the survivor group than in the non-survivor group (90.8% vs. 71.2%, *p* < 0.001). Among the patients who were treated, fluconazole was more frequently used in the survivor group (40.7% vs. 21.6%, *p* = 0.007) while echinocandins were more often employed in the non-survivor group (75.7% vs. 50.4%, *p* = 0.006). In addition, among 182 patients who were treated with the antifungal agent, the proportion of septic shock was non-significantly higher in the patients treated with echinocandins than those treated with non-echinocandins (42.2% vs. 28.8%, *p* = 0.071). Furthermore, there were significantly more patients with septic shock (60.6% vs. 19.3%, *p* < 0.001) and mycological failure (72.1% vs. 8.5%, *p* < 0.001) in the non-survivor group. The EQUAL Candida scores were lower in the non-survivor group with a higher proportion of patients with EQUAL Candida score < 15 for overall patients (52.9% vs. 37.0%, *p* = 0.017) and patients with the central venous catheter (44.6% vs. 26.9%, *p* = 0.020) as well as with EQUAL Candida score < 12 for patients without central venous catheter (38.1% vs. 14.6%, *p* = 0.045) (Table 2). The main differences regarding variables of the EQUAL Candida score between the survivor group and non-survivor group who had a central venous catheter were ophthalmology examination (survivor group, 62.7% vs. non-survivor group, 32.7%, *p* < 0.001), antifungal treatment for 14 days after first negative blood culture (survivor group, 45.8% vs. non-survivor group, 11.9%, *p* < 0.001), and central venous catheter removal ≤ 24 h from diagnosis of candidemia (survivor group, 79.7% vs. non-survivor group, 56.4%, *p* = 0.003). For patients who did not have a central venous catheter, the main differences regarding variables of the EQUAL Candida score between the survivor group and non-survivor group were ophthalmology examination (survivor group, 78.9% vs. non-survivor group, 44.0%, *p* = 0.004), and antifungal treatment for 14 days after first negative blood culture (survivor group, 36.8% vs. non-survivor group, 16.0%, *p* = 0.073).

**Figure 3:**
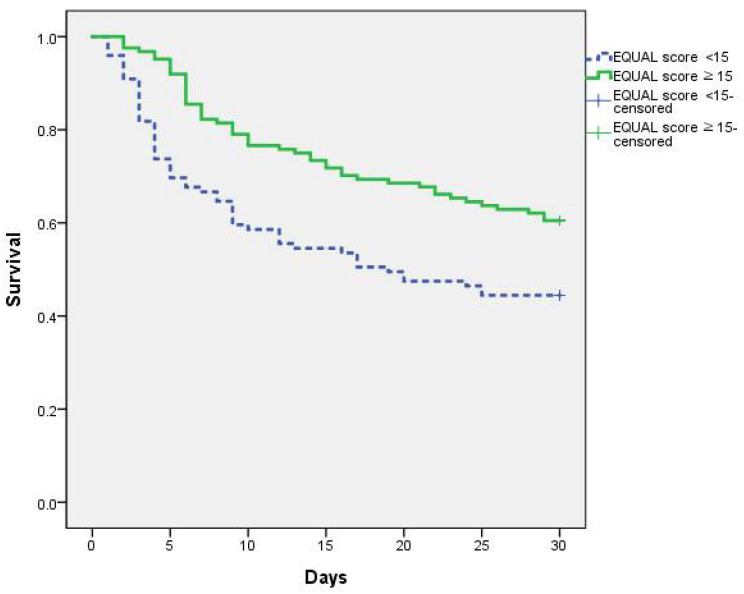
Kaplan–Meier survival curve stratified by the EQUAL Candida score ^1^ (^1^ EQUAL Candida score, The European Confederation of Medical Mycology Quality of Clinical Candidemia Management score).

Furthermore, the Kaplan–Meier curves with the log-rank test revealed that there was a significant difference in terms of 30-day survival after diagnosis of candidemia between patients with an EQUAL Candida score ≥ 15 (60.5%) and patients with an EQUAL Candida score < 15 (44.4%), *p* = 0.003 (Figure 3).

The multivariate logistic regression analysis was performed, which showed that a Charlson comorbidity index ≥ 4 (odds ratio [OR] 3.302, 95% confidence interval [CI] 1.276–8.546, *p* = 0.014), neutropenia (OR 7.855, 95% CI 1.669–36.963, *p* = 0.009), duration of hospital stay before candidemia diagnosis ≥ 21 days (OR 2.475, 95% CI 1.067–5.746, *p* = 0.035), septic shock (OR 4.242, 95% CI 1.710–10.524, *p* = 0.002), mycological failure (OR 29.519, 95% CI 11.175–77.970, *p* < 0.001), and EQUAL Candida score < 15 (OR 3.501, 95% CI 1.380–8.881, *p* = 0.008) were significantly associated with the 30-day mortality after diagnosis of candidemia. In addition, mechanical ventilation (OR 3.028, 95% CI 0.999–9.177, *p* = 0.050) and previous admission to ICU within three months (OR 2.726, 95% CI 0.999–7.437, *p* = 0.050) showed a borderline significance (Table 3).

### 3.3. Candidemia and Candida Species with Resistance Patterns

The most frequently isolated *Candia* species was *C. albicans* (41.7%), followed by *C. tropicalis* (23.3%), *C. parapsilosis* (20.6%), and *C. glabrata* (9.4%) for overall patients. Between period 1 and period 2, the proportion of non-*albicans Candida* was not significantly different (period 1: 60.9% vs. period 2: 57.1%, *p* = 602). However, there was a decrease in the proportion of *C. glabrata* in period 2 (5.8% vs. 17.4%, *p* = 0.006) while non-significant trends of an increase in the proportion of *C. parapsilosis* and *C. tropicalis* were observed in period 2. Although there were no reported cases of fluconazole resistance in period 1, the emergence of fluconazole resistance was noted in period 2 among *C. albicans*, *C. tropicalis*, *C. parapsilosis*, and *C. glabrata* isolates. Notably, there was a significant increase in fluconazole resistance among *C. parapsilosis* isolates in period 2 than in period 1 (35.3% vs. 0.0%, *p* = 0.020). These are shown in Table 4. There were no cases of caspofungin resistance among isolated *Candia* species. The minimum inhibitory concentration (MIC) 50 and MIC 90 values of the Candida species are shown in Supplementary Table S1.

## 4. Discussion

A significant increase in the incidence of candidemia over the study periods was observed in our study, which is consistent with a previous study [15] conducted in the ROK. Several studies identified risk factors for patients with candidemia in hospitals, which include older age, comorbidities, and medical conditions such as recent surgery, central venous catheter placement, indwelling urinary catheter use, parenteral nutrition, neutropenia, use of antibiotics, prolonged hospital stay, and mechanical ventilation [16,17,18,19]. In the present study, there were significantly more patients with older age, a longer duration of hospital stay before candidemia diagnosis, mechanical ventilation, parenteral nutrition, and urinary tract catheterization in period 2 than in period 1. Since these factors are considered to be risk factors for candidemia from previous studies [16,17,18,19], our findings suggest that an increased number of older debilitated patients with more severe illness in period 2 might contribute to an increased incidence of candidemia. As the patient population with aging and predisposing risk factors is expected to be increasing, the incidence of candidemia might also be predicted to rise. Thus, continued surveillance needs to be considered for an accurate estimate of the incidence of candidemia. Of note, there were more patients with others or unknown sources of candidemia in period 1 while there were more patients with urinary tract source of candidemia in period 2. This difference in terms of the source of candidemia between the study periods may be secondary to the patients’ clinical conditions and their possible underlying pathophysiology. The stress response caused by surgery may induce impaired wound healing and immune function, and possible translocation from the gut [20,21]. Moreover, candiduria is common among patients with a urinary tract catheter, and the majority of candiduria may represent contamination or colonization. However, candiduria may lead to candidemia [22]. As there was a higher prevalence of recent surgery, steroid use, and neutropenia in period 1 and urinary tract catheterization in period 2, this difference in patient characteristics might have had a differential influence on the development of candidemia. Our study showed that the use of antifungal agents for candidemia treatment was significantly different between the study periods, with more frequent use of echinocandins in period 2, which is consistent with a previous study [10] reported in the ROK. These findings reaffirm an increased echinocandin use for candidemia treatment after the approval of echinocandin use by the NHIS and publication of the guideline.

In our study, the 30-day mortality after diagnosis of candidemia was higher than that of a study reported in Japan [23]. As there were more patients with mechanical ventilation and septic shock in our study when compared to a Japanese study [23], the higher rate of 30-day mortality observed in our study might be due to an increased severity of candidemia in the cohort of study patients. Moreover, our comparison analysis showed that non-survivors had more mechanical ventilation, central venous catheter, hemodialysis, mycological failure, and septic shock when compared to survivors, suggesting the potential impact of disease severity on the 30-day mortality. Furthermore, there was more steroid use, neutropenia, longer duration of hospital stay before candidemia diagnosis, previous admission to ICUs within three months, and Charlson comorbidity index ≥ 4 in the non-survivor group than in the survivor group. These results are in agreement with previous studies [23,24,25,26] as comorbidities and clinical conditions that may affect immunity have been identified to be risk factors associated with mortality among candidemia patients. Prompt antifungal treatment is a critical component of candidemia management, as delaying antifungal treatment of candidemia has been associated with mortality [27]. Non-survivors who did not receive antifungal treatment had a significantly shorter length of hospital stay after diagnosis of candidemia than non-survivors who received antifungal treatment (median 3 days vs. 9 days, *p* < 0.001, data not shown) in our study. Extrapolating from these results and the higher proportion of septic shock in the non-survivors suggests that more severely ill candidemia patients with septic shock might have died before being considered for antifungal treatment in our study. Of note, there was the more frequent use of echinocandins in the non-survivors, which is in contrast to a previous study [28] reporting an association of an echinocandin treatment with decreased mortality. However, the proportion of septic shock was higher in the patients treated with echinocandins than those treated with non-echinocandins. Also, there were more septic shock, comorbidities, and clinical conditions that may affect immunity in the non-survivors, we speculate that these factors might have contributed to an increased risk of mortality in our study, rather than by echinocandins themselves. Furthermore, the multivariate logistic regression analysis revealed that a Charlson comorbidity index ≥ 4, neutropenia, duration of hospital stay before candidemia diagnosis ≥ 21 days, septic shock, and mycological failure were significantly associated with 30-day mortality after diagnosis of candidemia. These results further support the hypothesis mentioned above that septic shock, comorbidities, and clinical conditions that may affect immunity are considered to be significant predictors for mortality. The median EQUAL Candida score was lower in the non-survivors than that of the survivors in our study. Moreover, there was a significant difference in 30-day survival after diagnosis of candidemia between patients with an EQUAL Candida score ≥ 15 and patients with an EQUAL Candida score < 15. Additionally, an EQUAL Candida score < 15 was significantly associated with 30-day mortality after diagnosis of candidemia from the multivariate logistic regression analysis. These results are consistent with a previous study [11], which reported that greater guideline adherence with a higher EQUAL Candida score was associated with survival among patients with candidemia. Therefore, our results suggest that greater guideline adherence may be one of the critical components of candidemia management. Also, suboptimal compliance of the guideline with a lower EQUAL Candida score could be one of the predictors of mortality among candidemia patients.

Among isolated *Candia* species from candidemia patients, the proportion of non-*albicans Candida* was not significantly different between period 1 and period 2. These results were not in agreement with previous studies [6,7] of reporting an increase of non-*albicans Candida* candidemia recently. The possible reasons for this inconsistent observation may be due to potential differences in the local epidemiology of candidemia and the patient population, as the present study was conducted at a single center in the ROK. Nonetheless, trends of a non-significant increase in the proportion of *C. parapsilosis* and *C. tropicalis* were observed in period 2 in our study, which suggests the need for continued candidemia surveillance for the accurate evaluation of local epidemiology of candidemia. Of note, non-survivors had a higher proportion of *C. tropicalis* when compared to survivors, which is consistent with a previous study [29] reporting poor prognosis of *C. tropicalis* among non-*albicans Candida* candidemia. The possible tendency of *C. tropicalis* for mortality associated with *C. tropicalis* candidemia might be due to the virulence factors expressed by *C. tropicalis* species [30]. Regarding the trend of antifungal resistance, there was an increase of fluconazole resistance among isolated *Candida* species in period 2 when compared to period 1, particularly for *C. parapsilosis* isolates. These results may indicate a major change in candidemia epidemiology. In line with our results, recent studies [31,32] reported the emergence of fluconazole resistance of *C. parapsilosis* isolates in intensive care units. Besides, a mutation of the ERG11 gene in fluconazole-resistant *C. parapsilosis* isolates from candidemia patients was reported from a recent study conducted in the ROK [33]. Of note, the majority (76.7%) of patients with molecular mutation and fluconazole resistance had no antifungal exposure within 30 days prior to candidemia detection [33]. Furthermore, mutation of ERG11 or combined mutation of other genes (e.g., MDR1 gene) has been associated with fluconazole resistance in *C. albicans* and *C. tropicalis* isolates [34,35]. Taken together, our results of a higher rate of fluconazole resistance among *Candida* species in period 2 might have been due to possible clonal transmission of the fluconazole-resistant mutation genes. Subsequently, such clonal transmission of the fluconazole-resistant mutation genes might have led to the nosocomial spread of fluconazole-resistant *Candida* species.

Our study has some limitations, mainly due to a retrospective single-center study design with a relatively small sample size. Therefore, there might have been risks of potential confounding effects from unmeasured variables on our analyses. For example, we did not measure the rate of urinary catheter removal in candidemia patients diagnosed with urinary tract related candidemia. Also, we did not measure the exact dosing of antifungal agent used for candidemia treatment, which made it difficult to assess the possibility of under dosing of antifungal agent, particularly for fluconazole. Thus, these might have had possible effects on the treatment outcomes. In addition, specific fungal blood culture bottles with a dedicated fungal culture medium which may have higher sensitivity for detecting candidemia [36] was not available at our institution. Thus, the incidence of candidemia might have been underestimated during the study period. Moreover, we did not examine the genetic mutation of isolates of *Candida* species to test our hypothesis of their possible contribution to the emergence of fluconazole resistance in period 2. Of note, the proportion of *C. glabrata* was decreased in period 2, and there were no cases of caspofungin resistance among isolates of *Candida* species in our study. These results contrast to a recent study [37], which showed an increasing trend of *C. glabrata* with echinocandin resistance. These contradictory findings might be due to potential differences in the clinical setting and patient population. Additionally, the duration of our study periods might not have been long enough to reflect the details of the changing epidemiology of candidemia. Thus, future prospective studies with the inclusion of more centers and more extended study periods may be required for further assessment of the changing epidemiology of candidemia.

## 5. Conclusions

An increase in the incidence of candidemia and fluconazole resistance of the isolated *Candida* species was observed during the study periods of recent years 2013–2018. In addition, disease severity, comorbidities, and lower adherence to the candidemia guideline were associated with mortality among hospitalized adult patients. Therefore, our results highlight the need for continued surveillance of candidemia epidemiology and improvement in the adherence to the candidemia guideline.

## Figures and Tables

**Table 1:** Demographic and clinical characteristics of the candidemia patients stratified by study period 1 (2013–2015) and period 2 (2016–2018).

	Total	Period 1	Period 2	*p*-Value
	N = 223 (%)	(2013–2015)	(2016–2018)	
		N = 69, (%)	N = 154, (%)	
Age, median (IQR ^1^)	71 (60–79)	67 (57–76)	72 (64–80)	0.027
Age ≥ 75 years	85 (38.1)	18 (26.1)	67 (43.5)	0.013
Male	127 (57.0)	46 (66.7)	81 (52.6)	0.05
Female	96 (43.0)	23 (33.3)	73 (47.4)	
Main comorbidities				
Charlson comorbidity index, median (IQR)	3 (1–6)	3 (2–6)	3 (1–6)	0.97
Diabetes mellitus	83 (37.2)	24 (34.8)	59 (38.3)	0.614
Malignancy	113 (50.7)	39 (56.5)	74 (48.1)	0.242
Chronic central nervous system disease	64 (28.7)	16 (23.2)	48 (31.2)	0.223
Chronic kidney disease	72 (32.3)	19 (27.5)	53 (34.4)	0.31
Chronic liver disease	28 (12.6)	11 (15.9)	17 (11.0)	0.307
Chronic pulmonary disease	30 (13.5)	9 (13.0)	21 (13.6)	0.905
Chronic heart disease	103 (46.2)	36 (52.2)	67 (43.5)	0.23
Clinical conditions				
Mechanical ventilation	66 (29.6)	13 (18.8)	53 (34.4)	0.019
Urinary catheter	166 (74.4)	41 (59.4)	125 (81.2)	0.001
Central venous catheter	160 (71.7)	47 (68.1)	113 (73.4)	0.42
Parenteral nutrition	207 (92.8)	60 (87.0)	147 (95.5)	0.045
Hemodialysis	46 (20.6)	11 (15.9)	35 (22.7)	0.247
Steroid use	98 (43.9)	40 (58.0)	58 (37.7)	0.005
Neutropenia	19 (8.5)	10 (14.5)	9 (5.8)	0.032
Chemotherapy	60 (26.9)	18 (26.1)	42 (27.3)	0.854
Duration of hospital stay before candidemia diagnosis median days, (IQR)	20 (8–41)	13 (6–33)	21 (9–45)	0.046
Recent surgery in the current admission	61 (27.4)	32 (46.4)	29 (18.8)	<0.001
Previous admission to intensive care unit within 3 months	84 (37.7)	21 (30.4)	63 (40.9)	0.136
Previous use of antibiotics within 1 month	200 (89.7)	64 (92.8)	136 (88.3)	0.313
Source of candidemia				
Gastrointestinal tract	26 (11.7)	6 (8.7)	20 (13.0)	0.356
Central venous catheter	130 (58.3)	35 (50.7)	95 (61.7)	0.125
Urinary tract	12 (5.4)	0 (0.0)	12 (7.8)	0.02
Abscess	3 (1.3)	0 (0.0)	3 (1.9)	0.554
Others or unknown	52 (23.3)	28 (40.6)	24 (15.6)	<0.001
Antifungal treatment				
No antifungal treatment	41 (18.4)	17 (24.6)	24 (15.6)	0.107
Antifungal treatment	182 (81.6)	52 (75.4)	130 (84.4)	
Fluconazole	60/182 (33.0)	29/52 (55.8)	31/130 (23.8)	<0.001
Voriconazole	1/182 (0.5)	1/52 (1.9)	0/130 (0.0)	0.286
Amphotericin B	5/182 (2.7)	4/52 (7.7)	1/130 (0.8)	0.024
Echinocandins ^2^	116/182 (63.7)	18/52 (34.6)	98/130 (75.4)	<0.001
Antifungal treatment duration, median days (IQR)	13 (5–16)	11 (6–16)	13 (5–16)	0.885
Length of hospital stay after diagnosis of candidemia, median days (IQR)	19 (7–34)	13 (6–33)	19 (7–35)	0.734
EQUAL Candida score ^3^				
For overall patients, median (IQR)	15 (14–17)	14 (12–17)	16 (14–18)	<0.001
For patients with central venous catheter ^4^ (IQR)	17 (14–18)	15 (14–17)	17 (14–18)	0.001
For patients without central venous catheter ^5^ (IQR)	14 (12–15)	12 (11–14)	14 (12–15)	0.04
Mortality day 30 after diagnosis of candidemia	104 (46.6)	30 (43.5)	74 (48.1)	0.527

^1^ IQR, interquartile range, ^2^ Echinocandins including micafungin, caspofungin, and anidulafungin, ^3^ EQUAL Candida score, The European Confederation of Medical Mycology Quality of Clinical Candidemia Management score, ^4^ For patients with central venous catheter, data calculated for 48 patients for period 1 and 113 patients for period 2, ^5^ For patients without central venous catheter, data calculated for 21 patients for period 1 and 41 patients for period 2.

**Table 2:** Comparison analysis of the candidemia patients for risk factors for 30-day mortality.

	Total N = 223 (%)	Survivor N = 119, (%)	Non-Survivor N = 104, (%)	*p*-Value
Age, median (IQR ^1^)	71 (60–79)	71 (60–79)	72 (59–79)	0.725
Age ≥ 75 years	85 (38.1)	46 (38.7)	39 (37.5)	0.859
Male Female	127 (57.0) 96 (43.0)	68 (57.1) 51 (42.9)	59 (56.7) 45 (43.3)	0.951
Main comorbidities				
Charlson comorbidity index, median (IQR)	3 (1–6)	3 (1–5)	3 (1–6)	0.078
Charlson comorbidity index ≥ 4	91 (40.8)	42 (35.3)	49 (47.1)	0.073
Diabetes mellitus	83 (37.2)	45 (37.8)	38 (36.5)	0.844
Malignancy	113 (50.7)	55 (46.2)	58 (55.8)	0.155
Chronic central nervous system disease	64 (28.7)	36 (30.3)	28 (26.9)	0.584
Chronic kidney disease	72 (32.3)	34 (28.6)	38 (36.5)	0.204
Chronic liver disease	28 (12.6)	12 (10.1)	16 (15.4)	0.233
Chronic pulmonary disease	30 (13.5)	15 (12.6)	15 (14.4)	0.691
Chronic heart disease	103 (46.2)	59 (49.6)	44 (42.3)	0.277
Clinical conditions				
Mechanical ventilation	66 (29.6)	24 (20.2)	42 (40.4)	0.001
Urinary catheter	166 (74.4)	83 (69.7)	83 (79.8)	0.086
Central venous catheter	160 (71.7)	78 (65.5)	82 (78.8)	0.028
Parenteral nutrition	207 (92.8)	108 (90.8)	99 (95.2)	0.200
Hemodialysis	46 (20.6)	15 (12.6)	31 (29.8)	0.002
Steroid use	98 (43.9)	43 (36.1)	55 (52.9)	0.012
Neutropenia	19 (8.5)	3 (2.5)	16 (15.4)	0.001
Chemotherapy	60 (26.9)	26 (21.8)	34 (32.7)	0.069
Duration of hospital stay before candidemia diagnosis median days, (IQR)	20 (8–41)	12 (5–28)	29 (15–49)	<0.001
Duration of hospital stay before candidemia diagnosis ≥ 21 days	111 (49.8)	44 (37.0)	67 (64.4)	<0.001
Recent surgery in the current admission	61 (27.4)	36 (30.3)	25 (24.0)	0.299
Previous admission to intensive care unit within 3 months	84 (37.7)	36 (30.3)	48 (46.2)	0.014
Previous use of antibiotics within 1 month	200 (89.7)	106 (89.1)	94 (90.4)	0.748
Source of candidemia				
Gastrointestinal tract	26 (11.7)	15 (12.6)	11 (10.6)	0.638
Central venous catheter	130 (58.3)	62 (52.1)	68 (65.4)	0.045
Urinary tract	12 (5.4)	7 (5.9)	5 (4.8)	0.723
Abscess	3 (1.3)	2 (1.7)	1 (1.0)	1.000
Others or unknown	52 (23.3)	33 (27.7)	19 (18.3)	0.096
Candida species of candidemia *C. albicans* *C. parapsilosis* *C. tropicalis* *C. glabrata* *C. krusei* *C. guilliermondii* *C. utilis* Other *Candida* species ^2^	93 (41.7) 46 (20.6) 52 (23.3) 21 (9.4) 2 (0.9) 4 (1.8) 2 (0.9) 3 (1.3)	49 (41.2) 32 (26.9) 20 (16.8) 14 (11.8) 2 (1.7) 0 (0.0) 2 (1.7) 0 (0.0)	44 (42.3) 14 (13.5) 32 (30.8) 7 (6.7) 0 (0.0) 4 (3.8) 0 (0.0) 3 (2.9)	0.864 0.013 0.014 0.199 0.500 0.046 0.500 1.000
Antifungal treatment No antifungal treatment Antifungal treatment	41 (18.4) 182 (81.6)	11 (9.2) 108 (90.8)	30 (28.8) 74 (71.2)	<0.001
Fluconazole Voriconazole Amphotericin B Echinocandins ^3^	60/182 (33.0) 1/182 (0.5) 5/182 (2.7) 116/182 (63.7)	44/108 (40.7) 1/108 (0.8) 3/108 (2.8) 60/108 (50.4)	16/74 (21.6) 0/74 (0.0) 2/74 (2.7) 56/74 (75.7)	0.007 1.000 1.000 0.006
Septic shock Mycological failure	86 (38.6) 85 (38.5)	23 (19.3) 10 (8.5)	63 (60.6) 75 (72.1)	<0.001 <0.001
EQUAL Candida score ^4^ For overall patients, median (IQR) EQUAL score < 15 for overall patients	15 (14–17) 99 (44.4)	15 (14–18) 44 (37.0)	14 (14–17) 55 (52.9)	0.222 0.017
For patients with central venous catheter ^5^ (IQR)	17 (14–18)	17 (14–18)	16 (14–17)	0.052
EQUAL score < 15 for patients with central venous catheter ^5^	58 (36.0)	21 (26.9)	37 (44.6)	0.020
For patients without central venous catheter ^6^ (IQR)	14 (12–15)	14 (12–15)	14 (11–14)	0.074
EQUAL score < 12 for patients without central venous catheter ^6^	14 (22.6)	6 (14.6)	8 (38.1)	0.054

^1^ IQR, interquartile range, ^2^ Other *candida* species including *C. sphaerica*, *C. haemulonii*, and *C. lustaniae*, ^3^ Echinocandins including micafungin, caspofungin, and anidulafungin, ^4^ EQUAL Candida score, The European Confederation of Medical Mycology Quality of Clinical Candidemia Management score, ^5^ For patients with central venous catheter, data calculated for 78 patients for the survivor group and 83 patients for the non-survivor group, ^6^ For patients without central venous catheter, data calculated for 41 patients for the survivor group and 21 patients for the non-survivor group.

**Table 3:** Factors associated with the 30-day mortality after diagnosis of candidemia.

	Odds Ratio	95% Confidence Interval	*p*-Value
Charlson comorbidity index ≥ 4	3.302	1.276–8.546	0.014
Neutropenia	7.855	1.669–36.963	0.009
Duration of hospital stay before candidemia diagnosis ≥ 21 days	2.475	1.067–5.746	0.035
Septic shock	4.242	1.710–10.524	0.002
Mycological failure	29.519	11.175–77.970	<0.001
EQUAL Candida score ^1^ < 15	3.501	1.380–8.881	0.008
Mechanical ventilation	3.028	0.999–9.177	0.050

^1^ EQUAL Candida score, The European Confederation of Medical Mycology Quality of Clinical Candidemia Management score.

**Table 4:** Candida species of candidemia with fluconazole susceptibility stratified by study period 1 (2013–2015) and period 2 (2016–2018).

	Total N = 223 (%)	Period 1 (2013–2015) N = 69, (%)	Period 2 (2016–2018) N = 154, (%)	*p*-Value
*C. albicans* Fluconazole susceptibility	93 (41.7) 87/93 (93.5)	27 (39.1) 27/27 (100.0)	66 (42.9) 6/60 (90.9)	0.602 0.176
*C. parapsilosis* Fluconazole susceptibility	46 (20.6) 34/46 (73.9)	12 (17.4) 12/12 (100.0)	34 (22.1) 22/34 (64.7)	0.424 0.020
*C. tropicalis* Fluconazole susceptibility	52 (23.3) 51/52 (98.1)	15 (21.7) 15/15 (100.0)	37 (24.0) 36/37 (97.3)	0.709 1.000
*C. glabrata* Fluconazole susceptibility ^1^	21 (9.4) 19/20 (95.0)	12 (17.4) 11/11 (100.0)	9 (5.8) 8/9 (88.9)	0.006 0.450
*C. krusei* Fluconazole susceptibility	2 (0.9) 0/2 (0.0)	0 (0.0) NA ^2^	2 (1.3) 0/2 (0.0)	1.000 NA
*C. *guilliermondii** Fluconazole susceptibility	4 (1.8) 4/4 (100.0)	1 (1.4) 1/1 (100.0)	3 (1.9) 3/3 (100.0)	1.000 NA
*C. utilis* Fluconazole susceptibility	2 (0.9) 2/2 (100.0)	0 (0.0) NA	2 (1.3) 2/2 (100.0)	1.000 NA
Other *Candida* species ^3^ Fluconazole susceptibility ^4^	3 (1.3) 2/2 (100.0)	2 (2.9) 1/1 (100.0)	1 (0.6) 1/1 (100.0)	0.227 NA

^1^ Fluconazole susceptibility of *C. glabrata* available for 11 cases in period 1 and 9 cases in period 2, ^2^ NA, not available, ^3^ Other *candida* species including *C. sphaerica* and *C. haemulonii* in period 1 and *C. lustaniae* in period 2, ^4^ Fluconazole susceptibility available for 1 case in period 1 (*C. haemulonii*) and 1 case (*C. lustaniae*) in period 2.

## Data Availability

The data presented in this study are available on request from the corresponding author.

## Supplementary Materials

The following are available online at https://www.mdpi.com/article/10.3390/jof7040275/s1, Supplementary Table S1. MIC50 and MIC90 values of the *Candida* species during the study periods.

## References

[1] Pappas P.G., Kauffman C.A., Andes D.R., Clancy C.J., Marr K.A., Ostrosky-Zeichner L., Reboli A.C., Schuster M.G., Vazquez J.A., Walsh T.J. (2016). Clinical Practice Guideline for the Management of Candidiasis: 2016 Update by the Infectious Diseases Society of America. Clin. Infect. Dis..

[2] Horn D.L., Neofytos D., Anaissie E.J., Fishman J.A., Steinbach W.J., Olyaei A.J., Marr K.A., Pfaller M.A., Chang C.H., Webster K.M. (2009). Epidemiology and outcomes of candidemia in 2019 patients: Data from the prospective antifungal therapy alliance registry. Clin. Infect. Dis..

[3] Antinori S., Milazzo L., Sollima S., Galli M., Corbellino M. (2016). Candidemia and invasive candidiasis in adults: A narrative review. Eur. J. Intern Med..

[4] Guinea J. (2014). Global trends in the distribution of Candida species causing candidemia. Clin. Microbiol. Infect..

[5] Morii D., Seki M., Binongo J.N., Ban R., Kobayashi A., Sata M., Hashimoto S., Shimizu J., Morita S., Tomono K. (2014). Distribution of Candida species isolated from blood cultures in hospitals in Osaka, Japan. J. Infect. Chemother..

[6] Jung S.I., Shin J.H., Song J.H., Peck K.R., Lee K., Kim M.N., Chang H.H., Moon C.S., Korean Study Group for Candidemia (2010). Multicenter surveillance of species distribution and antifungal susceptibilities of Candida bloodstream isolates in South Korea. Med. Mycol..

[7] Yoo J.I., Choi C.W., Lee K.M., Kim Y.K., Kim T.U., Kim E.C., Joo S.I., Yun S.H., Lee Y.S., Kim B.S. (2009). National surveillance of antifungal susceptibility of Candida species in South Korean hospitals. Med. Mycol..

[8] Woo E.K., Han C., Jo S.A., Park M.K., Kim S., Kim E., Park M.H., Lee J., Jo I. (2007). Morbidity and related factors among elderly people in South Korea: Results from the AnsanGeriatric (AGE) cohort study. BMC Public Health.

[9] Hsu L.Y., Lee D.G., Yeh S.P., Bhurani D., Khanh B.Q., Low C.Y., Norasetthada L., Chan T., Kwong Y.L., Vaid A.K. (2015). Epidemiology of invasive fungal diseases among patients with haematological disorders in the Asia-Pacific: A prospective observational study. Clin. Microbiol. Infect..

[10] Choi H., Kim J.H., Seong H., Lee W., Jeong W., Ahn J.Y., Jeong S.J., Ku N.S., Yeom J.S., Kim Y.K. (2019). Changes in the utilization patterns of antifungal agents, medical cost and clinical outcomes of candidemia from the health-care benefit expansion to include newer antifungal agents. Int. J. Infect. Dis..

[11] Huang H.Y., Lu P.L., Wang Y.L., Chen T.C., Chang K., Lin S.Y. (2020). Usefulness of EQUAL Candida scores for predicting outcomes in patients with candidemia: A retrospective cohort study. Clin. Microbiol. Infect..

[12] Singer M., Deutschman C.S., Seymour C.W., Shankar-Hari M., Annane D., Bauer M., Bellomo R., Bernard G.R., Chiche J.D., Coopersmith C.M. (2016). The Third International Consensus Definitions for Sepsis and Septic Shock (Sepsis-3). JAMA.

[13] Mellinghoff S.C., Hoenigl M., Koehler P., Kumar A., Lagrou K., Lass-Flörl C., Meis J.F., Menon V., Rautemaa-Richardson R., Cornely O.A. (2018). EQUAL Candida Score: An ECMM score derived from current guidelines to measure QUAlity of Clinical Candidaemia Management. Mycoses.

[14] Bassetti M., Vena A., Meroi M., Cardozo C., Cuervo G., Giacobbe D.R., Salavert M., Merino P., Gioia F., Fernández-Ruiz M. (2020). Factors associated with the development of septic shock in patients with candidemia: A post hoc analysis from two prospective cohorts. Crit. Care.

[15] Park J.S., Cho S.H., Youn S.K., Bak Y.S., Yu Y.B., Kim Y.K. (2016). Epidemiological Characterization of Opportunistic Mycoses between the Years 2006 and 2010 in Korea. J. Microbiol. Biotechnol..

[16] Conde-Rosa A., Amador R., Pérez-Torres D., Colón E., Sánchez-Rivera C., Nieves-Plaza M., González-Ramos M., Bertrán-Pasarell J. (2010). Candidemia distribution, associated risk factors, and attributed mortality at a university-based medical center. Puerto Rico Health Sci. J..

[17] Li D., Xia R., Zhang Q., Bai C., Li Z., Zhang P. (2017). Evaluation of candidemia in epidemiology and risk factors among cancer patients in a cancer center of China: An 8-year case-control study. BMC Infect. Dis..

[18] Karabinis A., Hill C., Leclercq B., Tancrède C., Baume D., Andremont A. (1988). Risk factors for candidemia in cancer patients: A case-control study. J. Clin. Microbiol..

[19] Ortíz Ruiz G., Osorio J., Valderrama S., Álvarez D., Elías Díaz R., Calderón J., Ballesteros D., Franco A. (2016). Risk factors for candidemia in non-neutropenic critical patients in Colombia. Med. Intensiva..

[20] Finnerty C.C., Mabvuure N.T., Ali A., Kozar R.A., Herndon D.N. (2013). The surgically induced stress response. JPEN J. Parenter. Enter. Nutr..

[21] Deitch E.A., Bridges R.M. (1987). Effect of stress and trauma on bacterial translocation from the gut. J. Surg. Res..

[22] Storfer S.P., Medoff G., Fraser V.J., Powderly W.G., Dunagan W.C. (2014). Candiduria: Retrospective Review in Hospitalized Patients. Infect. Dis. Clin. Pract..

[23] Ishikane M., Hayakawa K., Kutsuna S., Takeshita N., Ohmagari N. (2019). The impact of infectious disease consultation in candidemia in a tertiary care hospital in Japan over 12 years. PLoS ONE.

[24] Vaquero-Herrero M.P., Ragozzino S., Castaño-Romero F., Siller-Ruiz M., Sánchez González R., García-Sánchez J.E., García-García I., Marcos M., Ternavasio-de la Vega H.G. (2017). The Pitt Bacteremia Score, Charlson Comorbidity Index and Chronic Disease Score are useful tools for the prediction of mortality in patients with Candida bloodstream infection. Mycoses.

[25] Kang S.J., Kim S.E., Kim U.J., Jang H.C., Park K.H., Shin J.H., Jung S.I. (2017). Clinical characteristics and risk factors for mortality in adult patients with persistent candidemia. J. Infect..

[26] Schroeder M., Weber T., Denker T., Winterland S., Wichmann D., Rohde H., Ozga A.K., Fischer M., Kluge S. (2020). Epidemiology, clinical characteristics, and outcome of candidemia in critically ill patients in Germany: A single-center retrospective 10-year analysis. Ann. Intensive Care.

[27] Morrell M., Fraser V.J., Kollef M.H. (2005). Delaying the empiric treatment of candida bloodstream infection until positive blood culture results are obtained: A potential risk factor for hospital mortality. Antimicrob. Agents Chemother..

[28] Andes D.R., Safdar N., Baddley J.W., Playford G., Reboli A.C., Rex J.H., Sobel J.D., Pappas P.G., Kullberg B.J., Mycoses Study Group (2012). Impact of treatment strategy on outcomes in patients with candidemia and other forms of invasive candidiasis: A patient-level quantitative review of randomized trials. Clin. Infect. Dis..

[29] Ko J.H., Jung D.S., Lee J.Y., Kim H.A., Ryu S.Y., Jung S.I., Joo E.J., Cheon S., Kim Y.S., Kim S.W. (2019). Poor prognosis of Candida tropicalis among non-albicans candidemia: A retrospective multicenter cohort study, Korea. Diagn. Microbiol. Infect. Dis..

[30] Negri M., Silva S., Henriques M., Oliveira R. (2012). Insights into Candida tropicalis nosocomial infections and virulence factors. Eur. J. Clin. Microbiol. Infect. Dis..

[31] Thomaz D.Y., de Almeida J.N., Lima G.M.E., Nunes M.O., Camargo C.H., Grenfell R.C., Benard G., Del Negro G.M.B. (2018). An Azole-Resistant Candida parapsilosis Outbreak: Clonal Persistence in the Intensive Care Unit of a Brazilian Teaching Hospital. Front. Microbiol..

[32] Pinhati H.M., Casulari L.A., Souza A.C., Siqueira R.A., Damasceno C.M., Colombo A.L. (2016). Outbreak of candidemia caused by fluconazole resistant Candida parapsilosis strains in an intensive care unit. BMC Infect. Dis..

[33] Choi Y.J., Kim Y.J., Yong D., Byun J.H., Kim T.S., Chang Y.S., Choi M.J., Byeon S.A., Won E.J., Kim S.H. (2018). Fluconazole-Resistant Candida parapsilosis Bloodstream Isolates with Y132F Mutation in ERG11 Gene, South Korea. Emerg. Infect. Dis..

[34] Fan X., Xiao M., Zhang D., Huang J.J., Wang H., Hou X., Zhang L., Kong F., Chen S.C., Tong Z.H. (2019). Molecular mechanisms of azole resistance in Candida tropicalis isolates causing invasive candidiasis in China. Clin. Microbiol. Infect..

[35] Paul S., Kannan I., Mohanram K. (2019). Extensive ERG11 mutations associated with fluconazole-resistant Candida albicans isolated from HIV-infected patients. Curr. Med. Mycol..

[36] Zheng S., Ng T.Y., Li H., Tan A.L., Tan T.T., Tan B.H. (2016). A dedicated fungal culture medium is useful in the diagnosis of fungemia: A retrospective cross-sectional study. PLoS ONE.

[37] Astvad K.M.T., Johansen H.K., Røder B.L., Rosenvinge F.S., Knudsen J.D., Lemming L., Schønheyder H.C., Hare R.K., Kristensen L., Nielsen L. (2018). Update from a 12-Year Nationwide Fungemia Surveillance: Increasing Intrinsic and Acquired Resistance Causes Concern. J. Clin. Microbiol..

