# Supplementary material for: Epidemiological Trends of Candidemia and the Impact of Adherence to the Candidemia Guideline: Six-Year Single-Center Experience
Source: J Fungi (Basel). 2021 Apr 6;7(4):275. doi: 10.3390/jof7040275 (PMC8067511; doi:10.3390/jof7040275)
Supplement: Supplementary file 1 [file jof-07-00275-s001.pdf]

Supplementary Table S1. MIC50 and MIC90 values of the *Candia* species during the study periods.

	C. albicans (n=93)		*C. tropicalis* (n=52)		*C. parapsilosis* (n=46)		*C. glabrata* (n=21)		*C. krusei* (n=2)	
	MIC50/MIC90	Range	MIC50/MIC90	Range	MIC50/MIC90	Range	MIC50/MIC90	Range	MIC50/MIC90	Range
Fluconazole	≤1 / 4	≤1-32	≤1 / ≤1	≤1-32	≤1 / ≥64	≤1-≥64	2 / ≥64	≤1-≥64	≤1 / 32	≤1-32
Voriconazole	≤0.12 / ≤1	≤0.12-8	≤0.12 / ≤1	≤0.12-32	≤0.12 / ≤1	≤0.12-≥8	0.25 / ≥8	≤0.12-≥8	≤0.12 / ≤0.12	≤0.12
Casfofungin	≤0.25 / ≤0.25	≤0.12-0.5	≤0.25 / ≤0.25	≤0.12-0.5	0.5 / 2	≤0.12-≥4	≤0.25 / ≤0.25	≤0.12-≤0.25	≤0.25 / ≤0.25	≤0.25
Mycafungin	≤0.06 / 0.5	≤0.06–0.5	≤0.06 / 0.5	≤0.06-2	0.5 / 2	≤0.06-2	≤0.06 / 0.5	≤0.06-0.5	≤0.06 / 0.12	≤0.06-0.12
Amphotericin B	≤0.25 / 0.5	≤0.06-2	≤0.25 / 0.5	≤0.25-≥4	0.5 / 0.5	≤0.25-1	≤0.25 / 1	≤0.25-1	0.5 / 0.5	0.5

Abbreviation: MIC50, concentration at which 50% of the isolates were inhibited; MIC90, concentrations at which 90% of the isolates were inhibited; MIC50/MIC 90 (μg/mL), Range (μg/mL)
